# Current challenges of alternative proteins as future foods

**DOI:** 10.1038/s41538-024-00291-w

**Published:** 2024-08-15

**Authors:** Yuwares Malila, Iyiola O. Owolabi, Tanai Chotanaphuti, Napat Sakdibhornssup, Christopher T. Elliott, Wonnop Visessanguan, Nitsara Karoonuthaisiri, Awanwee Petchkongkaew

**Affiliations:** 1 National Center for Genetic Engineering and Biotechnology (BIOTEC), National Science and Technology Development Agency (NSTDA), Khong Luang, Pathum Thani Thailand; 2 International Joint Research Center on Food Security (IJC-FOODSEC), Khong Luang, Pathum Thani Thailand; 3 School of Food Science and Technology, Faculty of Science and Technology, Thammasat University, Khong Luang, Pathum Thani Thailand; 4 Faculty of Biology, University of Cambridge, Cambridge, UK; 5 University of Chicago, Chicago, IL USA; 6 Institute for Global Food Security, School of Biological Science, Queen’s University Belfast, Belfast, UK

**Keywords:** Agriculture, Metabolomics

## Abstract

Global demand for food is expected to nearly double by 2050. Alternative proteins (AP) have been proposed as a sustainable solution to provide food security as natural resources become more depleted. However, the growth and consumer intake of AP remains limited. This review aims to better understand the challenges and environmental impacts of four main AP categories: plant-based, insect-based, microbe-derived, and cultured meat and seafood. The environmental benefits of plant-based and insect-based proteins have been documented but the impacts of microbe-derived proteins and cultured meat have not been fully assessed. The development of alternative products with nutritional and sensory profiles similar to their conventional counterparts remains highly challenging. Furthermore, incomplete safety assessments and a lack of clear regulatory guidelines confuse the food industry and hamper progress. Much still needs to be done to fully support AP utilization within the context of supporting the drive to make the global food system sustainable.

## Introduction

With the global population expected to reach 9.7 billion by 2050^1^, the world is now facing the challenge of producing sufficient and nutritious food to feed all at a time of increased competition and scarcity of land, water, and energy resources^2^. Among the three major macronutrients, the predicted demand for proteins has sparked a range of concerns, most notably whether its supply can be met by harvesting from traditional sources of protein alone, such as livestock. In addition, the livestock supply chain has been considered a significant contributor of greenhouse gases (GHG), accounting for approximately 14.5% of total GHG emissions; there is an association between livestock GHG emission and climate change^3^. With increasing demand for both human and animal feed, the conversion of forests, wetlands, and natural grasslands into agricultural lands is a threat to our environment and climate^4^. Such practices will lead to a marked loss of biodiversity^4^. Moreover, the rise in antibiotic resistance has been linked to the overuse and misuse of antibiotics in intensive animal production^4,5^.

**Fig. 1:**
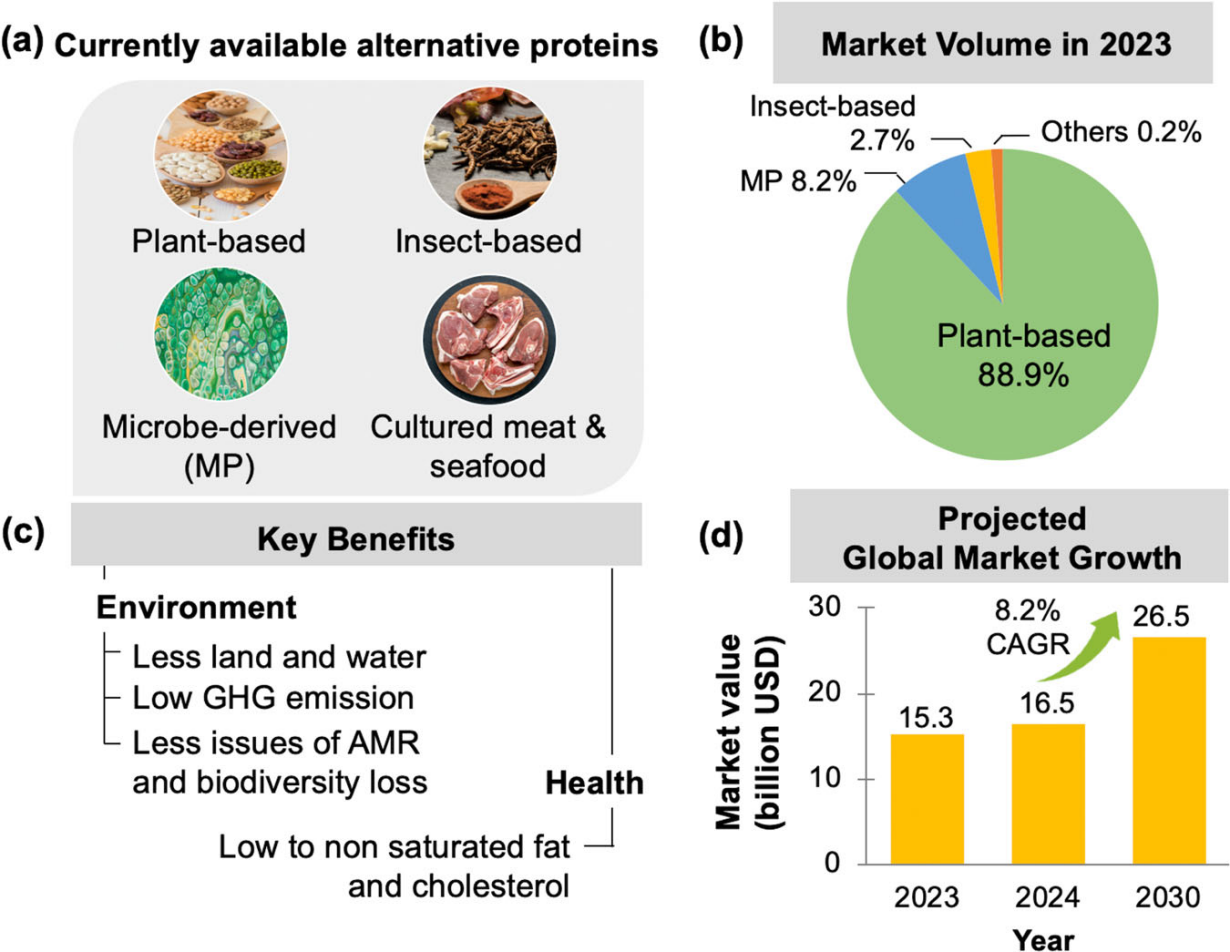
Overview of market interest and benefits of alternative protein (AP). **a** Currently available AP, **b** market volume, **c** key benefits, and **d** projected market growth of alternative proteins. MP refers to microbe-derived protein. GHG refers to greenhouse gases. AMR refers to antimicrobial resistance. CAGR refers to the compounded annual growth rate indicating a rate of return for an investment on the products. Pictures of each protein are designed by Freepik. https://www.freepik.com/free-photo/high-angle-beans-arrangement-concept_9861472.html; https://www.freepik.com/free-photo/emerald-green-bubbles-acrylic-painting_5682750.htm; https://www.freepik.com/free-photo/close-view-yummy-exotic-dish_5007103.htm. https://www.freepik.com/free-photo/top-view-fresh-meat-slices-raw-meat-round-wooden-desk-dark-food-freshness-animal-cow-meal-food-kitchen_17232551.htm.

In response to the global demands and concerns around future protein needs, the concept of alternative proteins (APs) was initiated and rapidly gained considerable attention^4^. This concept is not new as APs were predominantly and traditionally consumed by vegans and vegetarians, with soy, legumes, and wheat protein acting as the main ingredients^6^. Insects are also included in the diet as nutrient sources in some regions such as Africa and Asia but not on a regular basis^7,8^. However, there has recently been an upsurge of innovation backed by huge investments in the development of AP products with visual appearance and texture that are similar to animal-based products^4^. Protein sources have been diversified from a primarily livestock-based system to include four additional categories: plant-based, insect-based, microbe-derived, and cultured meat and seafood (Fig. 1). Each of these protein sources is at a different stage in its development. Plant-based proteins are the most widely accepted, as reflected by a markedly growing market segment^9^. It is predicted that plant-based proteins will be the first AP that achieves price parity with their conventional counterparts, followed by microbe-derived protein and cultured meat^10^. The AP market has been estimated to grow exponentially from USD 15.3 billion in 2023 to USD 26.5 billion by 2030 (Fig. 1)^11^.

Although APs have gained substantial interest amongst the scientific community as well as the food industry, they are still considered a small sector with a 42% decline in investment in 2022^12^; APs are not widely appealing to many consumers. This is mainly due to their sensory characteristics, relatively high prices, consumer unfamiliarity, and unassured health consequences^9,13–15^.

**Fig. 2:**
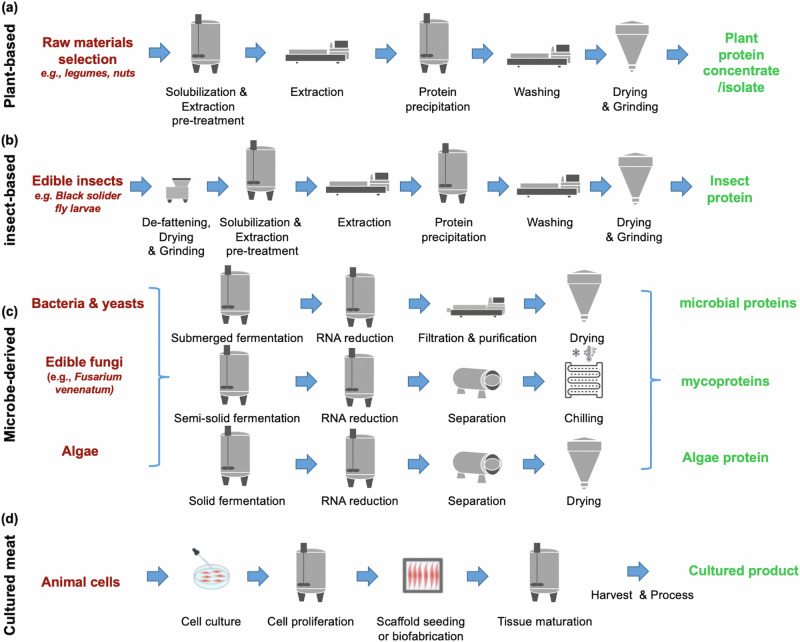
Production process diagram of alternative proteins. **a** plant-based protein, **b** insect-based protein, **c** microbe-derived proteins, and **d** cultured meat and seafood.

Today, the AP industry is facing significant challenges in terms of cost-effective scale-up production^16^. Compared to animal-based proteins, most of APs must be extracted, isolated, or separated from their starting materials (Fig. 2). Therefore, APs inherently exhibit different functional properties that play important roles in food processing technologies, so-called techno-functional properties. Therefore, it is challenging to formulate food products with similar properties and quality as seen in conventional products. In addition, the long-term health consequences of a dietary shift towards APs remain to be fully elucidated. A majority of consumers associate insect-based proteins and cultured meat with not being ‘natural’, feelings of disgust, and often refuse to even try the products^13^. Indeed, consumers appear to be more open to those alternatives if the products are proven to have global and societal benefits^13^. Regardless, the true environmental impacts of APs must be fully assessed and clearly communicated to consumers. Most importantly, there are still no international guidelines and standards for APs^17^, and the regulatory status varies among countries. For instance, during manuscript preparation, Singapore and the USA are the only two countries that recently approved the sale of cultured meat^18^, while other countries do not have any similar standard guidelines. There is little doubt that the combination of all these issues has markedly slowed the growth and utilization of APs.

The objective of this review is to systematically analyze the peer-reviewed scientific literature on the four categories of APs in terms of environmental impacts, challenges to utilization, future trends, and opportunities. The primary selection criteria include the published years between 2010 and 2023, the type of documents (i.e., original research and review article), and the search keywords (i.e., plant-based protein, plant-based meat analog, meat analog, insect protein, microbial protein, single-cell proteins, culture meat, lab-grown meat, cultivated meat and seafood). The reports with any risks of misleading results due to study design or conduct were excluded. Other materials, including news, regulations, standards, guidelines, and statistical information were further looked up for to complement the main context. Obtaining balanced and improved insights is needed for scientists, the food industry, and policymakers to support the future development of APs to better support global food security.

## Environmental impacts of APs

The potential environmental benefits of APs have been widely cited to promote foods based on these commodities^7–9,19–21^. Most studies focused on comparing the impacts between the production of livestock and APs such as plant farming, insect rearing, and microbial biomass fermentation. However, full life cycle assessment studies on the entire AP supply chain are very limited^22,23^.

The cultivation of plants, insects, and microorganisms generates less environmental burden than livestock farming. Growing high-protein plant materials (i.e., nuts, peas, and other pulses) emits 40 to almost 100 times less GHG (kg CO_2-eq_/100 g protein) and occupies 20–50 times less land space (m^2^/100 g protein) than lamb and bovine rearing (Table 1). Compared to pork, poultry, and farmed fish, plant cultivation produces 10–15 times less GHG but requires a similar land space. Similar benefits have also been observed for insects. For instance, it has been shown that mealworm (*Tenebrio molitor*) farming expels 75 times less GHG emission, occupies 102 times less land use, and produces 35 times less water footprint than those for ruminants^20^ (Table 1). In addition, insect farming generates markedly less methane and ammonia^21^, due to a greater efficiency for feed conversion^21^ than conventional livestock production. As for microbe-derived proteins, studies have shown up to 62 times lower GHG with almost 2000 times less land than animal-based proteins (Table 1). It is very interesting and indeed relevant that comparing the production of plant-based proteins with microbe-derived proteins, the latter consumes less land, by 3–80 times per 100 g protein, depending on the type of plants and the final products generated^24^. Another important point is that various forms of agro-industrial waste, such as pea-processing byproducts^25^, brewer-spent grains, and grape bagasse^26^, can be used as feed for the cultivation of filamentous fungi.

**Table 1:** Environmental impacts^a^

Source	Products	GHG emission		Land use		Water footprint	
Animal-based	Lamb & mutton	20	^169^	185	^169^	154	^170^
	Beef	50	^169^	164	^169^	/	
	Pork	7.6	^169^	11	^169^	5.9	^170^
	Poultry meat	5.7	^169^	7.1	^169^	4.3	^170^
	Fish (farmed)	6	^169^	3.7	^169^	0.4	
	Crustaceans (farmed)	18	^169^	2.0	^169^	/	
	Eggs	4.2	^169^	5.7	^169^	/	
	Cheese	11	^169^	39.8	^169^	/	
	Milk	3.2	^169^	9.0	^169^	/	
Plant-based	Nuts	0.3	^169^	7.9	^169^	/	
	Peas	0.4	^169^	3.4	^169^	/	
	Other pulses	0.5	^169^	7.3	^169^	/	
	Plant-based meat analogs (e.g., patties, nuggets, sausages)	0.7–3.1	^56^	0.3–2.2	^56^	/	
	Oat milk	0.04–0.08	^171^	0.11	^171^	/	
	Soy milk	0.006–0.04	^171^	0.01	^171^	/	
	Almond milk	0.07–0.08	^171^	0.07	^171^	/	
Insect-based	Mealworms (Netherlands)	0.3	^21^	1.8	^21^	4.3	^170^
	Crickets (Thailand)	0.4	^7^	/		0.42	^7^
	*Protaetia brevitarsis seulensis* larvae (Korea)	1.6	^172^	−0.2	^172^	/	
microbe-derived	Autotrophic hydrogen-oxidizing bacteria	0.8–1.2	^24^	0.1	^24^	/	
	Microbe-derived meat analog	1.0	^56^	0.1–1.5	^56^	/	
	Myoprotein meat analog (Quorn®)	2.2–5.9	^56^	0.4–1.1	^56^	/	
Cultured meat	cultured meat (assuming 26% protein)	8.7	^8^	/		/	

^a^greenhouse gas (GHG) emission in the unit of kg CO_2-eq_/100 g protein, land use in the unit of m^2^/100 g protein, and water footprint in the unit of m^3^/kg product.

The available literature indicates a marked decline in GHG emissions and land use for producing plant-based milk alternatives than cow’s milk (Table 1). Data has also shown that manufacturing meat analogs either from plant- or microbe-derived proteins emitted GHG at a greater quantity than growing the plants alone. Yet, it is considerably lower than that of animal-based products (Table 1). Production of alternative products from both plant and microbe-derived proteins requires less space than livestock-based products (Table 1). Data on the water footprints of these products require further investigation. It is worth noting that the current technologies for plant and insect-based protein extraction using alkaline solvent consume a large amount of energy and result in excess wastewater that requires further treatment. Technology-assisted extraction, product formulation, and the structuring of meat analogs are actively being developed; an assessment of the environmental impacts of APs must be regularly updated and the data made visible to all stakeholders.

The true environmental impacts of cultured meat and seafood have not been fully assessed yet as their production systems are still in their early phase. Only a few products (i.e., cultured chicken) have been commercialized in Singapore and the USA^27^. The process of culturing meat from cell culture and other agricultural products from cell cultures is known as cellular agriculture^28^. In terms of cultured meat, stem cells are grown in a media containing all the required nutrients (e.g., hormones, growth factors, and fetal bovine serum) under controlled conditions to enable cell division and proliferation in a conventional manner, such as the one seen in the vaccine industry. A recent study indicates that the carbon footprint and energy consumption of cultured meat depend greatly on the efficiency of the medium usage^27,29,30^. In the case of low efficiency, the carbon footprint and energy consumption per kg of obtained products could be as high as 22.6 kg CO_2-eq_ (8.7 kg CO_2-eq_/100 g protein, assuming equal protein content with beef) and 264 MJ respectively. If the culture medium was utilized at high efficiency, the carbon footprint and energy consumption could be theoretically reduced by 90% and 50% respectively^28^. However, this is far from being achievable given the current technology. Although the process of culturing meat requires less land^30–32^, the need for high concentrations of nutrients (e.g., glucose and amino acids) to culture the cells and tissues is expected to leave a negative impact on its environmental footprint^27^.

## Challenges in the application of APs in the food industry

### Technological challenges

Many of the AP products fail to fully mimic the organoleptic experience of their conventional counterparts and thus have received limited consumer acceptance^33^. Such technological challenges are based mainly upon molecular and physicochemical differences that cause substantial deviations in the techno-functional properties between alternative and animal proteins^34^.

Most plant proteins are globular and stored as preserved nutrients in storage organs (e.g., seeds and tubes)^35^, hence why protein extraction is required (Fig. 2a). In addition, plant proteins can form heat-induced gels and also exhibit emulsifying properties; however, a sufficiently large concentration is needed to achieve the same gel strength as that of animal-based proteins^36^. Soy and pea proteins show comparable solubility and emulsifying properties to egg proteins but a much higher temperature and longer heating time is required to obtain a similar texture to those of cooked eggs^34^. Although insect-based proteins can form heat-induced gels, they usually show poor foaming ability^37,38^. Insect proteins can be used in the form of whole insect flour or solvent-extracted insect-based proteins but the techno-functional properties of insect protein ingredients will be influenced by non-protein components (e.g., chitin)^36^. The structure and functional properties of insect proteins are very diverse, depending on the organ (e.g., muscular system vs cuticles), insect species, and stage of life^39^. Exposure to chemical solvents, mechanical milling, and thermal treatments during pretreatments and protein extraction (Fig. 2a, b) can induce partial denaturation and aggregation of plant- and insect-based proteins, altering techno-functional properties^34,40^.

The development of whole-muscle meat analogs is the most challenging aspect for plant- and insect-based proteins. Today, high-temperature extrusion is commonly used for producing commercial meat analogs. Extrusion technique can be classified based on moisture content into low-moisture (10–40%) and high-moisture (60–70%) extrusions. Low-moisture extrusion is a common process used for manufacturing texturized vegetable proteins. Meat analogs acquired from texturized vegetable proteins visually and texturally differ from animal meat^41^. On the contrary, high-moisture extrusion, by producing anisotropic structures resembling fibrous characteristics of whole-muscle meat^42^, is widely used in the production of commercial plant-based meat analogs and has also been shown to experimentally structure similar products from the insect-based^43^. Fat can be co-extruded to obtain fat marbling on meat cuts. However, the aggregates do not completely resemble the architecture of a muscle fiber, leading to different properties compared to conventional meat. Meat analogs are often perceived as lacking juiciness^44^, with low cohesiveness and a fibrous texture^45^. Considerable amounts of processing aids, e.g., structure-forming, flavoring, and coloring agents, are added to the products to compensate for the inferior techno-functional properties of plant- and insect-based proteins^46^. However, this practice does not align well with the growing trend of “clean label”. In addition, high-moisture extrusion is considered energy-intensive^42^. Process control of extrusion is challenging due to the complex interplay of process parameters and material properties^47^. Other developing technologies for whole-muscle structure formation include shear cell technology, electrospinning, 3D printing, and wet spinning^47^. Of these, shear cell technology, through its use of intensive shear and elevated temperatures to structure the proteins, has shown good potential for up-scaling operations^48^. However, these technologies remain a subject for more intensive examination. For other alternative products (e.g., milk, cream, butter yogurt, and eggs), the proteins are formulated as a single protein or protein blends with the inclusion of processing aids to produce gelling, emulsification, and foaming to increase stability during homogenization, thermal processes, and cold storage^49–51^.

Similar to plant- and insect-based proteins, the techno-functional properties of microbe-derived proteins are diverse, depending on the microbial species, production system, and non-protein components used^52^. Microbe-derived proteins are generally obtained from microbial biomass of bacteria, yeasts, fungi, and microalgae grown in bioreactors or other controlled environments (Fig. 2c)^53,54^. The biomass produced by the fermentation process is then harvested using centrifugation and may be subjected to protein extraction or other processing techniques. However, dry biomass exhibits inferior oil binding and foaming properties compared to the extracted microbe-derived proteins due to its high content of insoluble fiber^55^. In addition, thermal treatment, aimed at reducing RNA content in microbial cells, may induce partial denaturation of the proteins, thus altering its techno-functional properties^52^. Nonetheless, constructing meat analogs from filamentous fungi appears to be less challenging than plant- and insect-based proteins as the fiber-oriented structure, obtained after biomass centrifugation, already visually resembles ground meat^42^. The biomass can be dried and further formulated as chunks, incorporated into ground meat and comminuted products, or formed into whole-muscle meat using binding agents^42^. In terms of mycoproteins, while some commercial products have been marketed for several years, the available technology still greatly impacts the environment^56^.

**Fig. 3:**
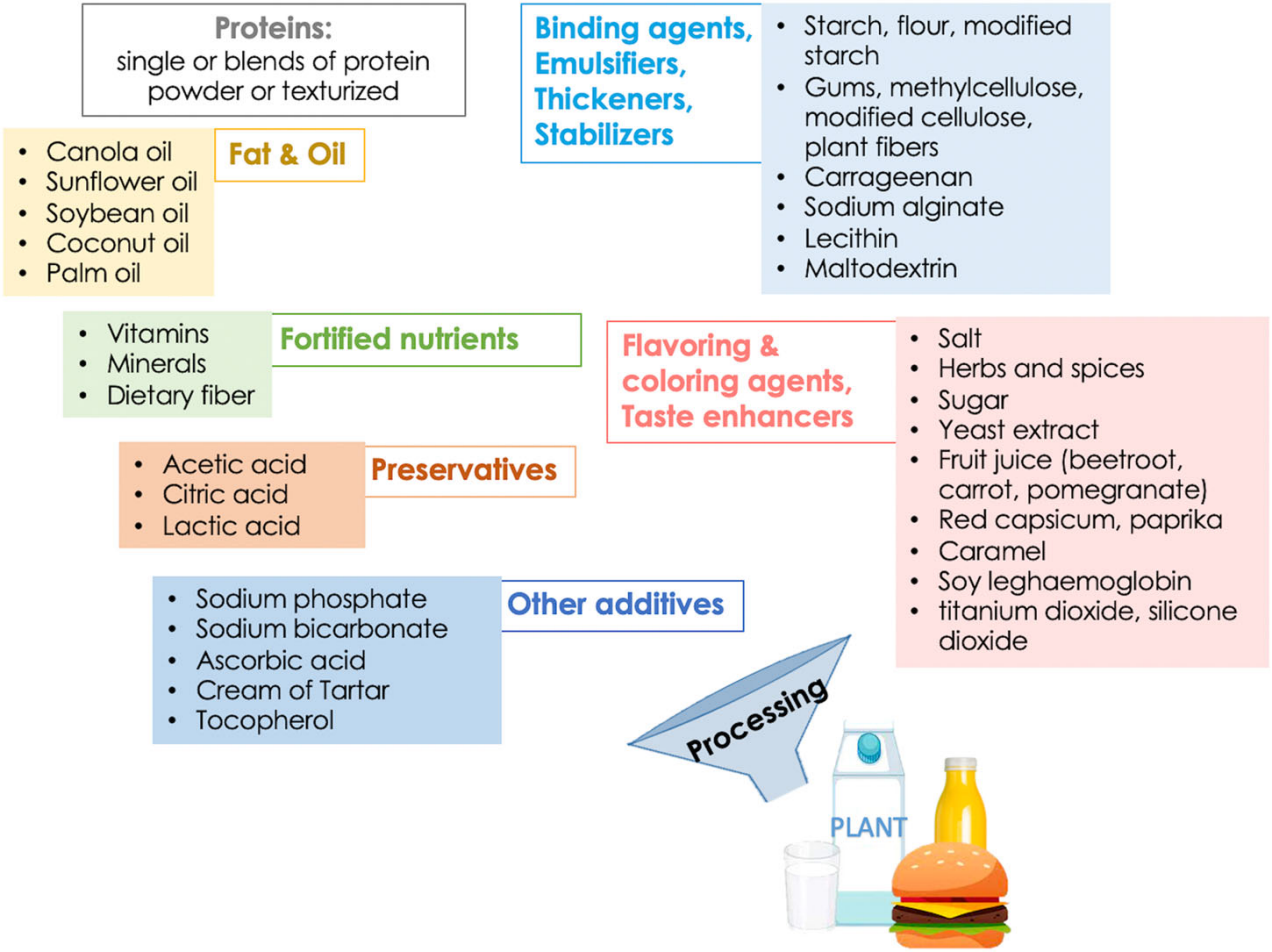
Ingredients in commercial alternative protein products. Common ingredients of current alternative proteins can be divided into seven groups, including source of proteins, texture enhancers (i.e., binding agents, emulsifiers, thickeners, and stabilizers), fat and oil, fortified nutrients, color and taste additives (i.e., flavoring agents, flavoring agents, taste enhancers), preservatives, and other additives. Pictures of food products are designed by Freepik. https://www.freepik.com/free-psd/detox-smoothie-drink-isolated_151238726.htm.

Another challenge of plant-based, insect-based, and microbe-derived proteins is mimicking the taste, odor, and flavor of animal-based products. Based on different biomolecules present, APs inherently have their own taste and flavor profiles that are very distinct from animal-based products. Plant-based proteins are often associated with beany and grassy flavors, a bitter and chalky taste, astringency, and a strong aftertaste^44^. The off-flavors in plants vary among plant cultivars and the amount of proteins used and may arise during protein extraction, food processing, and during storage^57^. For insects, the flavor profile is diverse, ranging from nutty, buttery, milky, earthy, to fishy^39,58^, depending on the species, stage of life, body part, and source of feed^39^. Some studies have tried to address the issue with the taste and flavor of edible insects^39,58^ but only a few have examined this in the actual insect-based proteins produced or in the final products^59,60^. The taste and flavor of microbial-based proteins are much less influenced by the type of substrates and the activities of enzymes released by the microbes. The exogenous enzymes further break down carbohydrates and proteins present in the substrate into sugars and peptides and free amino acids which govern the taste and flavor profile of the products. Fungi-based patties were described as salty, sour, bitter, astringent, and umami with a wide range of smells (e.g., nutty, fruity, and moldy)^52,61,62^. The off-notes of those APs may be reduced by using masking compounds^63^, but this remains challenging as little is known about the factors influencing the taste and flavor profiles of these proteins nor the molecular interactions among flavor and taste precursors during manufacturing processes and storage. Different proprietary processes from different manufacturers appear to result in unique off-flavor and off-taste characteristics; the strategy to combat this may involve uniquely designing and optimizing batch by batch^63^. Flavoring agents may be added (Fig. 3) to create a similar sensory profile to its animal-based counterparts, but the control and optimization of flavor blends require close attention^64^. Due to the aforementioned growing “clean label” trend, natural flavoring additives are now the preferred choice by consumers, but their stability and interaction within the new food matrix needs further evaluation. Ultimately, protein extraction processes from plants, insects, and microorganisms may need to be re-designed to yield proteins with neutral flavors.

In terms of cultured meat and seafood, the proteins are derived from actual stem cells of target animals and should, in theory, have fewer taste and flavor issues (Fig. 2d). The concept of this approach evolved around using mammalian and avian cells and tissues to replace land-based animals. The cellular agriculture approach has now been extended to fish, mollusk, and crustacean cells and tissues^32,64^. Production of cell-based seafood may be well-suited for scalable bioreactor cultivation relative to mammalian muscle tissues due to its unique physiological properties, including tolerance to hypoxia, high buffering capacity, and low-temperature growth conditions of aquaculture cells^65^. The cells are grown in bioreactors to obtain adequate cell quantities, and can then be harvested for ground and comminuted meat products when matured. Alternatively, the cells are anchored to edible scaffolds, e.g. texturized soy protein^66^, cellulose^67^, and salmon gelatin film^68^, to guide the development of three-dimensional (3D) multicellular structures for whole-muscle products. For the latter case, the lack of a circulatory system restricts the diffusion of nutrients and oxygen throughout the tissues^69^, complicating the culturing of full-sized whole-muscle meat cuts. The formation of a vascular network is also complex, time-consuming, and expensive^70^. Another potential means to form whole-muscle meat is to structure whole-muscle meat cuts using bioprinting^71,72^. Handral et al.^71^ used extrusion printing in which cells were extruded into filaments or fibers, but the shear stress generated may damage the cell membranes^73^. Later, the tendon-gel integrated bioprinting technique, proposed by Kang et al.^72^ was demonstrated to form a Wagyu steak by anchoring cell fibers on tendon gels. The cells had undergone differentiation, followed by assembling fibers and adipose tissue using transglutaminases. However, the steak obtained was still small (5 mm in diameter and 10 mm long). Techno-functional properties and sensory characteristics (i.e., color, texture, and flavor) of traditional meat proteins are strongly governed by muscle composition, thick and thin fiber arrangement, and post-mortem biochemical conversion of muscle to meat. In contrast, cultured cells predominantly contain embryonic or neonatal isoforms of actin and myosin^74^; the cultured tissues lack fat and collagenous connective tissue structure. Whether cultured meat exhibits a comparable texture to conventional meat still requires further evaluation. The idea of co-culturing several different cell types, such as myoblasts, fibroblasts, and adipocytes, has been proposed in an attempt to better mimic muscle composition. Nonetheless, because different cells grow and differentiate in specific media, changing media might induce sub-optimal conditions for one or more cell types^69^. The proposed technique along with information regarding the behavior of cultured tissues on texture and on the rate and extent of the tenderization process requires a more thorough investigation^69^. Production of further processed and comminuted meat products require sufficient protein content with functionality, particularly water-holding capacity, emulsifying, and gelling properties. At present, there is no scientific investigation describing the inherent techno-functional properties of cultured meat and seafood or the effects of further processing on their properties. Similar to other alternative products, processing aids could be added to compensate for incomplete protein functionality. Colorants can also be added to counteract discoloration^73^. As for visual color, cultured cells contain very low myoglobin content, giving cultured meat a colorless appearance. Color may be adjusted during cell culturing by directly adding pigments into the culture media^75^ or stimulating myoglobin expression^73^; however, more investigations are required. As for taste and flavor, it remains unclear whether the precursor compounds in cultured meat would be present in similar quantities as their conventional counterparts^73^. In addition, the absence of fat will impact flavor development during cooking^69^. Adding fat and flavoring agents during product manufacturing may help overcome these issues of cultured meat^69^, but further studies are required. Scaffold materials may also influence the development of the taste and flavor of the products.

The main technological issue of cultured meat and seafood is up-scaling. In Singapore, a commercial cultured meat company possesses a production capacity of 2–3 kg per week compared to the 4000–5000 metric tons of conventional chickens sold weekly in the country^76^. In the USA, an FDA-approved cultured meat company addressed its production size of cultured chicken meat at approximately 25 tons annually^77^ while approximately 2.9 million tons of chicken meats were produced nationally in 2021^78^. Large-scale production of cultured meat and seafood requires tightly controlled bioreactors, as slight variations in the growing conditions may cause massive production and contamination risks^42^. The currently available process is considered time and resource-intensive and is far from being economically viable^47^. Overall, the technology is far from being scalable or economically feasible.

### Nutritional profile

One of the benefits of APs have been associated with is health promotion. This is based upon the assumed reduced risk of chronic diseases brought by the consumption of red and processed meat, containing high saturated fat, cholesterol, and low fiber^79^. However, despite the well-documented benefits of plant-based diets for health^80–82^, critical evaluation of the effects of APs on health implications is still scarce and the fact that many are now classified under the heading of ‘ultra-processed foods’ is of substantial concern^83^. So far, consumers are still very hesitant to trust that such products are truly healthy and nutritious^84^.

In terms of AP products, recent studies have systematically analyzed the nutritional profiles of commercially available products based on their product nutrition labels (Table 2). Alternative products show lower energy density and saturated fat with higher dietary fiber than their animal-based counterparts. However, the products often contain more salt than their conventional counterparts^85–87^. Most plant-based products consist of lower protein content whereas insect-based and microbe-derived meat analogs show similar protein levels to their counterparts^88^. Commercial alternative products are often formulated based on extracted proteins along with other ingredients, including oils, flavoring, and coloring agents (Fig. 3) to create the visual, structure, and sensation of being derived from animals^84,89^. Based on their formulation, alternative products are often classified as ultra-processed foods^83^. Although a number of observational studies show a link between ultra-processed foods and diet-related adverse health effects^83^, further causational investigation is required to address this point. In addition, given that the health benefits of plant consumption are from biometabolites, (e.g., polyphenols), the extensive protein extraction and food processing may greatly influence their presence along with other bioactivities^89^. Hence, whether the alternative products are healthier diet choices than conventional animal-based proteins requires further scientific evidence.

**Table 2:** Key nutritional differences between alternative products and their animal-based counterparts

Source	Products		Research location	Key nutritional differences	Ref.
plant-based	Plant-based meat analog (*n* = 207)	vs meat products	UK retailers	- Lower energy density, saturated fat, and protein - High fiber and salt.	^85^
	Plant-based meat analog	vs meat products	German market	- Lower saturated fat, sodium, sugar, and overall calories	^173^
	Plant-based ground beef (*n* = 37)	vs ground beef	USA	- Lower saturated fat, protein, zinc, vitamin B12 - Higher salt - Good source of dietary fiber, iron, manganese, copper, folate, and niacin	^174^
	Plant-based burger (*n* = 7)	vs beef burger	USA	- Lower fat and saturated fat, bioavailability of protein, calcium, and iron - Similar or higher levels of protein - Higher levels of fiber, salt	^175^
	Plant-based steaks(*n* = 68)	vs meat products	Italy	- Lower saturated fat - Similar protein and total fat content, - Higher energy, total carbohydrates, sugar, and fiber	^176^
	Plant-based burgers (*n* = 105)	vs meat products		- Lower saturated fat, and sugar - Similar total fat, protein, and salt - Higher energy, total carbohydrates, sugar, and fiber	
	Plant-based meatballs (*n* = 22)	vs meat products		- Lower saturated fat, and protein - Similar total fat, and salt - Higher energy, total carbohydrates, sugar, and fiber	
	Plant-based cutlets (*n* = 34)	vs meat products		- Lower total fat, and saturated fat - Similar energy, protein, salt - Higher total carbohydrates, sugar, and fiber	
	Plant-based drinks	vs full-fat cow milk	Not specified	- Lower energy, protein (except for soy-based), and fat - Similar or higher carbohydrates	^96^
Insect-based	Insect burger patty (one brand)	vs beef patty	Not specified	- Lower saturated fat - Similar protein and fat content - Higher energy content, sugar, fiber, and sodium	^88^
Microbe-derived	Mycoprotein burger patties (one brand)	vs beef patty	Not specified	- Lower fat and saturated fat - Similar protein - Higher sugar, dietary fiber, and sodium - Slightly higher energy content	^88^
	Mycoprotein drink (one brand)	vs milk	UK	- Lower protein - Higher fat, carbohydrate, and fiber - Similar energy content	^177^
	Mycoprotein (one brand)	vs chicken meat	UK	- Higher energy, protein, fat, dietary fiber and carbohydrate	^99^

Considering the nutritional profile of each AP, plant proteins are often considered low in protein quality mainly due to their lower content of essential amino acids, particularly lysine and sulfur-containing amino acids compared to animal-based protein^90–92^. A previous study by Gorissen et al.^93^ reported essential amino acids content in commercially available plant-based protein isolates and found that only the products from soy (27% of total amino acid), brown rice (28%), pea (30%), corn (32%), and potato (37%) met the FAO/WHO requirements for essential amino acids. Oat (21% of total protein), lupin (21%), wheat (22%), and hemp (23%) were found to be below the requirement. Such problems could be overcome by blending different plant protein sources^94^. However, with the commercially available proteins, the target content of lysine along with isoleucine and leucine would be limiting constraints^94,95^. Plant-based drinks and formulas are not recommended as main liquid foods for children <1 years of age^96^. In the case of insect-based proteins, their essential amino acids appear to meet the FAO/WHO requirement^22^, however, the amino acid composition differs largely between species^97^, the origin of the insects, different feeding regimes, and methods of analyses employed^98^. As for microbe-derived proteins, the information regarding their nutritional profiles is still very limited. For mycoprotein, in most reported microbe-derived proteins, their amino acid profiles and essential amino acid contents were similar to those of chicken meat^99^ with protein digestibility corrected amino acid scores (PDCAAS) of 0.9^100,101^. However, these products show higher calories, protein, fat, dietary fiber, and carbohydrate than chicken meat^99^. It is worth noting that insects and mycoproteins comprise high chitin content^102^ which could lead to an overestimation of the protein concentration^103^. Even though cultured meat and seafood are grown in vitro from cultivated animal cells, their nutritional profiles remain to be elucidated. An investigation into the protein content, amino acid profiles, and protein digestibility of cultured meat and seafood is required. The nutritional profile of the scaffold also needs to be taken into consideration^73^.

Animal-based products are also considered good sources of micronutrients, particularly vitamin B12, iron, zinc, and trace minerals. However, vitamin B12 is absent in plant-based products and the other minerals appear to be relatively low in plant-based alternatives^92^, microbe-derived^25^, and cultured meat^104^. These compounds can be fortified into the final products but the biological efficacy of added inorganic minerals under the formulated condition requires further investigation^91,103^. Potential enrichment of micronutrients in mycoprotein through supplementation in the growth medium is possible and remains to be examined^25^. As for cultured meat, mechanisms of micronutrient uptake into the cultured cells also remains to be elucidated^105^. Above all, further investigation on whether the fortified micronutrients through those technologies provide similar benefits in human health at different age ranges is necessary to make properly informed decisions.

### Safety: allergens and contaminants

A major concern of APs is whether the protein is safe for human consumption. Despite their long history of consumption, the behavior of plant-based and insect-based proteins under different manufacturing processes as novel ingredients may differ from their conventional uses. Heavy metals (e.g., lead, arsenic, mercury, and cadmium), pesticide residues, mycotoxins, and pathogenic microorganisms may be carried over from raw materials^106,107^ or from substrates in the case of microbe-derived proteins^108^. The main concerns for microbe-derived proteins are toxic metabolites produced by the microbes and contamination of undesirable microorganisms^109^. Little is known about the effects of processing on such contaminants.

Of all the potential health hazards, the issues of allergens are particularly concerning. Exposure to new forms of food produced using AP-focused techniques may introduce unknown allergenic responses among susceptible consumers. For plant-based proteins, nuts, soybeans, and wheat are among the well-documented eight most common food allergens^110^, but little is known about other emerging plant-based proteins, such as those derived from peas and mung beans. Safety prediction could be performed by comparing amino acid sequences of a novel protein against known allergens; however, the current allergen sequence databases are insufficient for proper and robust safety assessments^111^. Insect-based proteins also contain known allergenic compounds (e.g., arginine kinase, and tropomyosin) but their clinical significance remains to be elucidated^46^. In 2021, the EFSA Panel on Nutrition, Novel Foods and Food Allergens (NDA) raised the risk of allergy to yellow mealworm proteins, particularly among consumers who are allergic to crustaceans^112^. Despite the direct reports on the allergenicity of microbe-derived proteins, the cases of microbe-based foods suggest that the allergenic potent of the novel proteins requires close attention^113,114^. The risks also extend to microbe-derived recombinant protein analogs. Indeed, some of those precision-fermentation products, e.g., human milk oligosaccharides derived from modified *E. coli*, meat-flavor soy leghemoglobin produced by engineered *Komagataella phaffii*, and casein from engineered *Saccharomyces cerevisiae*, have received FDA approval to commercialized in the US^115^. Allergenicity risks of the precision-fermentation technology itself are recently underpinned by the Food Systems and Food Safety Division of FAO^116^. This is due to the lack of full comprehension of the metabolites of the modified microbes; whether they produce existing allergens, alter proteins to be allergenic, or novel compounds still not known as allergens^116^. Although many allergens are heat labile and can be inactivated during thermal processing, the allergenicity of some known allergens remains unaltered after thermal treatment^117–120^. This issue was observed for prolamin, the largest allergenic plant protein family, and tropomyosin, a major allergen in crustaceans and potentially insects^120^. In addition, research describing the effects of thermal processing on the allergenicity of the novel proteins is still limited. Above all, these APs are used in food products in the form of protein isolates (70–95% protein), protein concentrates (60–70% protein), and protein hydrolysates (90–95% protein) which contain higher protein loads than their natural forms. Frequent exposure at a high concentration of those ingredients may trigger allergenic reactions in susceptible consumers^120^.

Plants and insects that are common sources of APs often carry antinutrients. Plant antinutrients, such as protease inhibitors (e.g., trypsin inhibitors), polyphenols (e.g., tannins), and phytates, generally interfere absorption and utilization of nutrients. In some cases, however, stronger effects may include a leaky gut, autoimmune diseases (e.g., lectins and some saponins), gut dysfunction, inflammation, and behavioral effects^121^. As for insects, the levels of antinutrients (e.g., hydrocyanide (2.2–3.2 mg/kg)), oxalate (13.2–28.4 mg/kg), phytate (0.3 mg/kg), and tannin (0.3–0.4 mg/kg) fall within acceptable limits^22^. However, the negative impacts of antinutrients are usually diminished after the ingredients are processed under appropriate processing conditions^46,120,122^.

Other potentially hazardous compounds can be formed when the proteins are processed at high temperatures^106^. Maillard reaction developed under dry heating was shown to result in the formation of new epitopes in wheat^123^ and nuts^124,125^. Further processing may result in the accumulation of advanced glycation end-products which are shown to increase the expression of pro-inflammatory cytokines in mammalian cells^126^. Glycation of pea protein with glucose and lactose could interfere with trypsin activity^127^, hence potentially reducing the bioavailability of proteins. More research is needed to elucidate this issue.

The main concerns for microbe-derived proteins are toxic metabolites produced by the microbes and contamination of undesirable microorganisms^128^. Mycotoxins are a group of toxic substances, produced by filamentous fungi, which are of concern for food safety, as they may cause harmful effects on the human organism and accumulate in the tissues of some animals. However, research on mycotoxins contamination in APs is still limited. The first study on mycotoxins occurrence and its exposure assessment in plant-based meat alternatives samples from Italy was conducted by Mihalache et al.^129^. Results showed that most of the samples (92%) were contaminated with at least one mycotoxin and mixtures of up to seven mycotoxins. Additionally, consumption of plant-based meat alternatives led to a non-tolerable exposure to alternariol (i.e., a toxic metabolite of Alternaria fungi) for vegan and vegetarian consumers, while samples contaminated with aflatoxins and ochratoxin A, indicated a health concern related to liver and renal cancer, respectively. Therefore, contamination with mycotoxins in APs, particularly plant-based protein alternatives, should be carefully monitored and controlled by producers and competent authorities.

For cultured meat and seafood, the current literature lacks a direct assessment with respect to food safety. In theory, they are less susceptible to microbial contamination, pesticides, and heavy metals relative to conventional animal-based meat^130^. However, potential hazards may arise from the source of the animal cells and cell culture media. There is the possibility that the cultured cells may carry zoonotic disease agents (e.g., parasites, viruses, pathogens, and infectious prions) and without a broad level screening of cell lines prior to ensure no infectious agents is present will present a substantial risk^130^. In addition, when cells are stressed, which is often the case in cell culture there is the potential for the release of histamine^130–132^. Some of those biological agents may be able to propagate or persist under certain processing conditions^133^. The accumulation of this in the final product is possible as histamine is known to be heat-resistant^134^. There are quite a large number of people who are intolerant to histamine and can suffer adverse health effects due to exposure^135^.

In the case that genetically modified cells are used, genomic stability particularly after continual sub-culturing for multiple passages must be assessed^136^. During cell cultivation, the concentration of any substances in the media, both intended (e.g., fetal bovine serum) and unintended (e.g., cell-secreted growth factors, and histamine) can accumulate, leading to high concentrations of those substances^130^. The biological response due to the consumption of those compounds at such levels is still not fully understood. In addition, whether chemical and structural changes of either the cells, scaffolds, and medium residues during food manufacturing affect human health requires further investigation^137^.

### Regulatory framework

The lack of clear and transparent standards and guidelines at both national and international levels regarding APs is considered one of the key issues slowing down progress in the industry. The most essential reason for the legislative framework is the assurance of the safety of consumers and fair trade among food companies across countries^138^. The regulatory frameworks must also include labeling of the products so that consumers can easily make their informed choices.

APs are likely to be regulated as novel foods, i.e., foods and food ingredients that do not have a history of safe use, and regulatory status regarding novel foods differs across countries. In the EU, the European Commission (EC) novel food legislation regulation (EC 2015/2283) has been issued and has been in force since 2018^139^. As for the USA, there is no specific definition or regulation for novel foods^138^. Instead, the US Food and Drug Administration (USFDA) considers any new food ingredients as food additives or generally recognizes as safe (GRAS) for specific intended use. USFDA has recently issued a draft guidance on “Labeling of Plant-Based Milk Alternatives (PBMA) and Voluntary Nutrient Statements” on February 2023 with the goal to assist PBMA producers in providing consumers with clear labeling^140^.

The regulatory status regarding novel foods in Asia is somewhat behind Europe and the USA. Only China, South Korea, Singapore, and Thailand have specific regulations for novel foods. The regulation of novel foods in China resembles the EU model^141^. As for South Korea, novel ingredients are registered under the Temporary Standards and Regulations for Food Ingredients (MFDS Notification No.2016–27). The record shows that in 2017, there were 20 cases that have been approved as novel ingredients, including insects, plants, microorganisms, and sweeteners^142^. In August 2023, South Korea issued a draft amendment that will legally enable plant-based meat alternatives to use the word “meat” on their labels^143^. Recently later, they have officially published the Standards for Recognition of Temporary Standards and Specifications for Food which recognizes cell and microbial culture as food ingredients^144^.

In contrast to less specific regulations among these countries with regard to novel foods, the Singapore Food Agency (SFA) and Thai Food and Drug Administration (FDA) have more specific safety assessment criteria and guidance on APs^145^. SFA requires a safety assessment of potential human health hazards of the novel ingredients, both in their most basic form as well as at the level of actual food products and conditions during consumption. In addition, it is the responsibility of companies to adopt effective testing methodologies in compliance with international standards (e.g., ISO/IEC 17025) based on the hazards that may be present in the novel foods. On the other hand, the Thai FDA requires a sub-chronic study or at the minimum a clinical research study in healthy people for safety assessment^145^.

## Future trends and opportunities

**Fig. 4:**
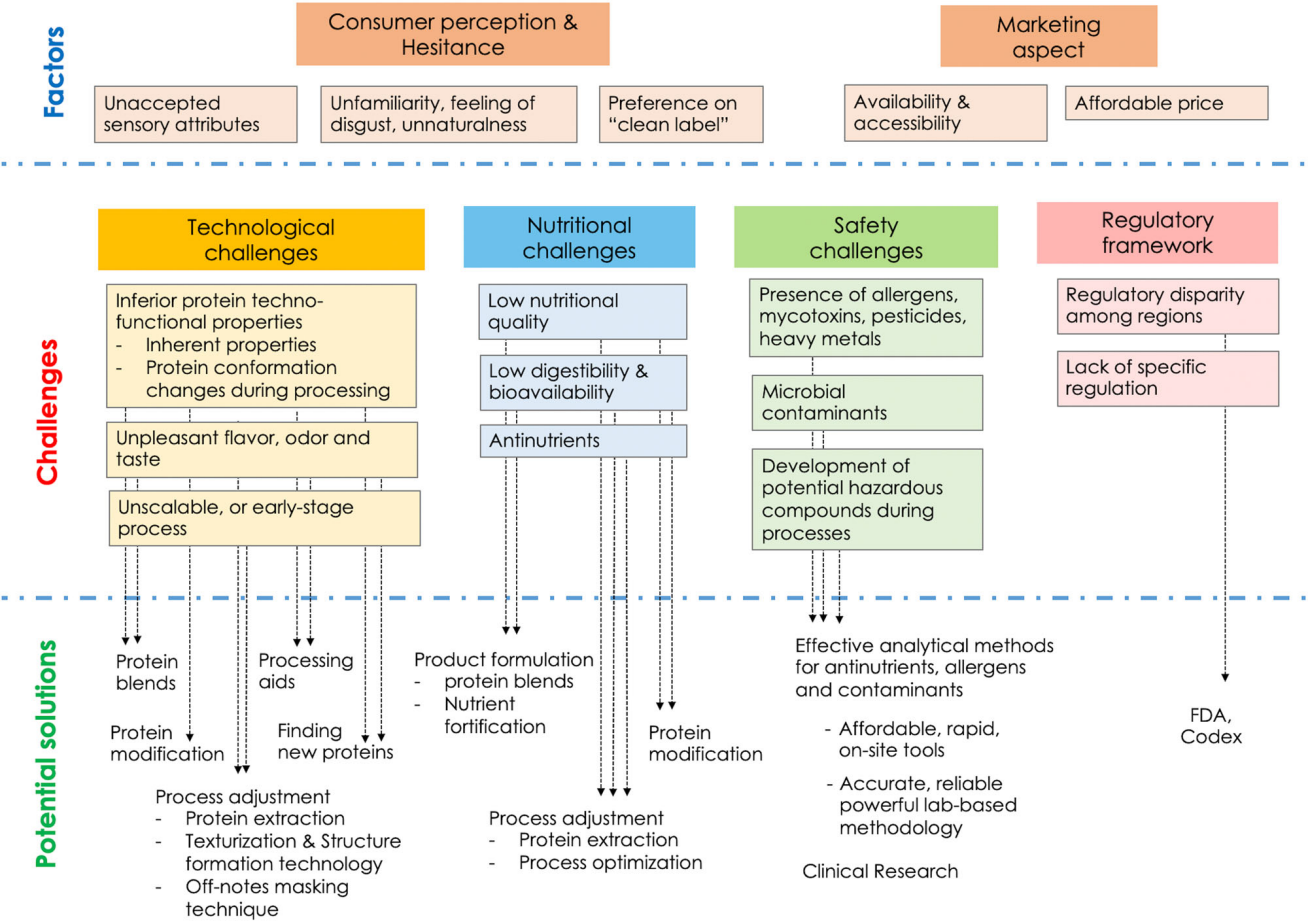
Challenges, influencing factors, and potential solutions for alternative proteins. Dat-line arrows connect between challenging issues of alternative proteins with their potential solutions.

The growing trend of APs opens opportunities for new protein sources as well as technological innovations (Fig. 4). The issues of nutritional quality, technological properties, and allergenicity of the currently used APs will serve to drive the search for diversifying protein sources. Algae, seaweed, and water plants are of interest as new protein sources due to their rapid growth, high protein content, and unique nutritional profiles. An example is *Wolffia* spp., an aquatic plant of the duckweed family, which offers nine essential amino acids^146^ with a PDCAAS score of 0.89^147^, dietary fiber, several trace minerals^148^, and bioactive vitamin B12^149^. Scientists and the food industry have been exploring potential uses of their indigenous plants such as Bambara groundnut, a pulse crop largely cultivated in West Africa^149–151^. The use of local produce may not only be favorable due to crop tolerance but also align with the aim to reduce carbon footprint during the export and import of non-local protein ingredients. Given a wide variety of APs accompanied by an increase in data about food, the formulation of protein blends can serve the nutritional demands of many^93,95^. In addition, oilseeds and vegetable byproducts, such as sunflower, potato, and chickpea, from food manufacturing can be upcycled to produce protein ingredients. Such practices not only help support food security but also the goal of having a sustainable global food supply system.

With diversifying protein sources comes better technology-assisted protein extraction and protein recovery, which can be developed further for maximum production efficiency^152^. An application of supercritical fluid extraction using CO_2_ and ethanol, for example, has been reported to remove the off-flavor compounds from pea protein isolates^153^. High-pressure processing has been shown to improve penetration of extraction solvent within the cell, hence increasing yield without altering protein functionalities^154^.

In terms of cultured meat and seafood, immortal cell lines have been developed through advanced gene-editing technology in order to increase proliferative capacity^155^. However, cell characteristics, metabolic activities, along with genetic stability, and scalability require further assessment. Cell culture medium, edible micro-carriers, and scaffolding materials are also under extensive investigation^155^. Fermentation has been one of the key technologies employed to produce ingredients for APs and will continue to play an important role in this industry^156^. This includes the use of precision-fermentation-based “cell factories” for the production of replacement ingredients for dairy and egg proteins^157^, pigments of meat analogs^158^, and the off-flavor masking agents for plant-based proteins^159^. The beneficial effects of fermentation on removing unpleasant beany off-flavors are under extensive investigation^158,160,161^, due to its high efficiency, reduced harm to the protein matrix, targeted performance, and low budget requirements^159^. In addition, the utilization of proper microorganisms can also improve the aroma profile of the final products^158,161^. Nevertheless, redesigns of bioreactors for food-grade fermentation, minimizing downstream purification, and maximizing value extraction from left-over microbial biomass are required for a techno-economically feasible approach. Cost-effective up-scaling production of APs and their components is essential to reduce the price of the final products to gain consumer attraction.

While most scientific studies primarily aim at technological improvements, more studies on the safety and health effects are required. Research regarding the reduction of allergenicity in APs is underway but much still needs to be understood. Enzymatic reactions, either based on enzymatic cross-linking by transglutaminase^162,163^ or protein hydrolysis^164,165^, have been shown to reduce the allergenicity of soy, wheat proteins, and peanut proteins. A different approach to understanding the effect of introducing APs to a broad population is to look at its association with various, distinct metabolic processes, rather than the disease itself. In addition, with food transformation towards APs, it is possible to cause a shift in the gut microbiome^166,167^, which could cause changes to the body’s physiology. The development of serum-free medium and edible scaffolds for cultured meat and seafood production is also being explored^168^. Furthermore, the need for effective and affordable analytical methods to detect and verify levels of allergens, toxins, and other contaminants in the finished food product is urgently required. Standards and guidelines, including labeling, for APs have been discussed by the FAO/WHO as well as adhering bodies in many countries, and are needed to ensure the safety of consumers to a growing number of new food products^116^.

## Data Availability

All relevant data are available from the authors upon a reasonable request.
